# Omicron incidence and seroprevalence among children in Montreal, Canada, in early 2023: final results from the longitudinal EnCORE serology study

**DOI:** 10.1017/S0950268824000797

**Published:** 2024-09-25

**Authors:** Katia Charland, Laura Pierce, Adrien Saucier, Marie-Ève Hamelin, Margot Barbosa Da Torre, Julie Carbonneau, Cat Tuong Nguyen, Gaston De Serres, Jesse Papenburg, Guy Boivin, Caroline Quach, Kate Zinszer

**Affiliations:** 1 Centre for Public Health Research, University of Montreal, Montreal, QC, Canada; 2 Research Centre of Quebec-Université Laval, Quebec City, QC, Canada; 3 Ministère de la santé et des services sociaux, Quebec City, QC, Canada; 4 National Institute of Public Health of Quebec, Quebec City, QC, Canada; 5 Montreal Children’s Hospital of the McGill University Health Centre, Montreal, QC, Canada; 6 Departments of Microbiology, Infectious Diseases & Immunology and of Pediatrics, University of Montreal, Montreal, QC, Canada

## Abstract

Since early 2022, routine testing for severe acute respiratory syndrome coronavirus 2 (SARS-CoV-2) based on symptoms and exposure history has largely ceased in Canada. Consequently, seroprevalence studies, particularly longitudinal studies, have become critical for monitoring the rate of incident SARS-CoV-2 infections and the proportion of the population with evidence of immunity. EnCORE is a longitudinal SARS-CoV-2 seroprevalence study comprising five rounds of serology testing from October 2020 to June 2023, in a sample of 2- to 17-year-olds (at baseline), recruited from daycares and schools in four neighbourhoods of Montreal, Canada. We report on SARS-CoV-2 incidence and seroprevalence among the 509 participants in the fifth and final round of the study. Seroprevalence of antibodies from either infection or vaccination was 98% (95 per cent confidence interval [CI]: 97, 99). The infection-acquired seroprevalence was 78% (95% CI: 73–82), and the incidence rate was 113 per 100 person-years (95% CI: 94–132), compared to the seroprevalence of 58% and the incidence rate of 133 per 100 person-years, respectively, in the fourth round of testing (mid–late 2022). Of the 131 participants newly seropositive for infection in Round 4, only 18 were seronegative for infection in Round 5 (median follow-up: 326 days).

## Introduction

As the pandemic has evolved, public health monitoring of severe acute respiratory syndrome coronavirus 2 (SARS-CoV-2) has come to rely on serological studies to provide data on infection incidence and the proportion of the population with antibodies from past infection or vaccination. The EnCORE study is a longitudinal seroprevalence study comprising five rounds of serology testing for infection-acquired and vaccine-induced antibodies, in a cohort of children from Montreal, recruited through daycares, elementary schools, and high schools. Data collection for rounds 1 to 5 started in October 2020, May 2021, November 2021, May 2022, and February 2023, respectively. The last two rounds took place during the Omicron-dominant period in Montreal. Here, we present the results from the fifth and final round of testing, providing estimates of the SARS-CoV-2 seroprevalence, the incidence rate of infections between Round 4 and Round 5, and the likelihood of a child newly seropositive in Round 4, being seronegative in Round 5.

## Methods

SARS-CoV-2 serostatus of the participants was determined from the analysis of mailed-in dried blood spot (DBS) samples. Enzyme-linked immunosorbent assay (ELISA) tests identified the levels of antibodies against three antigens: the receptor-binding domain from the spike protein, the spike protein, and the nucleocapsid protein. Seropositivity by vaccination and/or infection was defined as being seropositive to at least two antigens. Infection-acquired seropositivity was based on the results of all three antigens [1]. To be classified as infection-acquired seropositive, vaccinated participants required seropositivity for the nucleocapsid protein and at least one other antigen, while unvaccinated participants required seropositivity to any two antigens. Participant characteristics and vaccination status were determined via parent-reported questionnaires. See Zinszer et al. (2021–2023) for details on study design, laboratory testing, and recruitment [1–2].

Overall seroprevalence (seropositivity from vaccination and/or infection) was estimated for the full sample as the percentage of participants seropositive for at least two antigens. The infection-acquired seroprevalence was estimated for the full sample of participants and by participant characteristics. To allow for longitudinal comparison of infection-acquired seroprevalence statistics, we adjusted for loss to follow-up by inverse probability of censoring weighting (IPCW). In addition, we present the infection-acquired seroprevalence adjusted for assay sensitivity and specificity [3]. Multivariable regression was used to estimate adjusted prevalence ratios (relative risk) of infection-acquired seropositivity by participant characteristics using the method of Zou [4]. Participant characteristics of interest included age, sex at birth, vaccination status, annual household income, and parent identification as an ethnic or racial minority. Confounders were identified from directed acyclic graphs [1]. The household income variable had the largest percentage of missing data at 17%; all others had less than 4% of their data missing. Regressions were based on multiply imputed data by multivariate imputation by chained equations (MICE) and were corrected for loss to follow-up using IPCW.

The infection-acquired seroconversion rate was defined as the number of infection-acquired seropositive children in Round 5 that were seronegative in Round 4 divided by the sum of time at risk, in the sample. Time at risk was calculated as the length of time between the participant’s Round 4 and Round 5 DBS dates (or first positive rapid or polymerase chain reaction (PCR) test date, when available).

For the durability of the antibody response, we reported on the number of children who seroreverted between Round 4 and Round 5. Seroreversion was defined as a return to seronegativity in participants who were previously seropositive for infection. The sample was all newly seropositive participants in Round 4 followed from their Round 4 DBS date (or positive rapid antigen or polymerase chain reaction (PCR) date, when available) to their Round 5 DBS date.

## Results

DBS samples were collected from 509 participants and were dated February 2023 to June 2023. Seropositivity from either infection or vaccination was found in 502 of 509 participants (seroprevalence: 98% [95% CI: 97, 99]). Four hundred and one participants were seropositive for infection, so the overall infection-acquired seroprevalence was 79% (95% CI: 75, 82). The infection-acquired seroprevalence corrected for loss to follow-up and corrected for assay sensitivity and specificity was 78% (95% CI: 73, 82) and 87% (95% CI: 82, 91), respectively. Infection-acquired seroprevalence estimates by participant characteristics showed a 17% greater risk in participants whose parent identified as an ethnic or racial minority, a 19% greater risk in participants from households with an annual household income below 100,000$ CAD (below approximately the first sample tercile for income), and a 19% greater risk among unvaccinated participants (Table 1). Vaccination was less common in children whose parents identified as an ethnic or racial minority (minority: 67% vs. not a minority: 92%) and in households with lower annual income (<100 000$ CAD: 79% vs. ≥100 000 $CAD: 92% vs. missing income data: 88%). However, when restricting the analysis to vaccinated children, there remained an elevated seroprevalence in the ethnic or racial minority group (minority: 84% vs. non-minority: 77%) and lower-income group (<100 000$ CAD: 87% vs. ≥100 000 $CAD: 74% vs. missing income data: 77%). Unvaccinated children had a significantly higher infection-acquired seroprevalence, even though only 62 of 450 vaccinated participants received their last dose within 120 days of their DBS. One hundred and forty-two participants received their last dose at least 450 days before their DBS. There was little evidence of age group or sex differences in infection-acquired seroprevalence.

**Table 1.** EnCORE Round 5 infection-acquired seroprevalence adjusted for loss to follow-up and crude seroconversion rate per 100 person-years, by participant characteristics, February–June 2023

	Infection-acquired^e^ seroprevalence			Infection-acquired^e^ seroconversion	
	N (N imputed)	Seroprevalence^a^ (95% CI)	Adjusted prevalence ratio (95% CI)	*N*	Seroconversion rate (per 100 person-years) (95% CI)
Overall	509	78% (73, 82)	–	197	113 (94, 132)
Sex at birth					
Male	257	75% (70, 81)	ref	109	109 (83, 135)
Female	252	80% (75, 86)	1.1 (0.96, 1.2)	88	117 (89, 145)
Age, years					
2–4	35	75% (60, 90)	0.93 (0.75, 1.1)	8	90 (1.8, 178)
5–11	222	76% (70, 82)	0.94 (0.85, 1.0)	88	106 (78, 134)
12–18	252	81% (76, 86)	ref	101	120 (93, 147)
Racial or ethnic minority^b^					
No	433 (439)	76% (72, 81)	ref	178	112 (92, 132)
Yes	67 (70)	90% (83, 97)	1.2 (1.1, 1.3)	17	112 (46, 179)
Annual household income^c^ (CAD)					
<100,000$	126 (153)	89% (83, 95)	1.2 (1.1, 1.3)	36	136 (87, 185)
≥100,000$	299 (356)	73% (67, 78)	ref	123	106 (83, 130)
Vaccination^d^					
No	59	85% (73, 96)	1.2 (1.0, 1.4)	12	94 (24, 163)
Yes	450	76% (72, 81)	ref	185	111 (94, 134)

a With IPCWs.

b Parent identification as an ethnic or racial minority.

c Annual household income prevalence ratio adjusted for age group, sex at birth, education, and vaccination.

d Vaccinated with at least one dose 10 or more days before DBS; vaccination prevalence ratio adjusted for annual household income, age group, sex at birth, and education.

e Classification as infection-acquired seropositive: vaccinated participants must be seropositive for the nucleocapsid protein and at least one other antigen, while unvaccinated participants must be seropositive for any two antigens.

The infection-acquired seroconversion rate was 113 per 100 person-years (Table 1). For the number of children seroreverting from a Round 4 infection, the sample had 131 children and the length of follow-up from infection detection date to Round 5 ranged from 217 to 492 days (median: 326 days). Of the 131 children newly seropositive for infection in Round 4, only 18 were seronegative for infection in Round 5 (after a minimum of 7 months). Of these, six had been vaccinated during follow-up.

## Discussion

The EnCORE study is one of the few longitudinal SARS-CoV-2 serology studies in children and has contributed to the literature on the evolving incidence of infection and both vaccine-induced seroprevalence and infection-acquired seroprevalence in a population who, in many ways, experienced the pandemic differently than older age groups – often with milder symptoms, with lower rates of vaccination, and with incomplete adherence to non-pharmaceutical interventions [1–2]. In this fifth and final round of serology testing, we found that 98% were seropositive from either vaccination or infection and 78% were seropositive from infection. The intensity of infection incidence declined from 133 (per 100 person-years) in mid–late 2022 to 113 in early-mid 2023. Lower annual household income, identification as an ethnic minority, and unvaccinated status remained important determinants of infection-acquired seropositivity.

**Figure 1.**
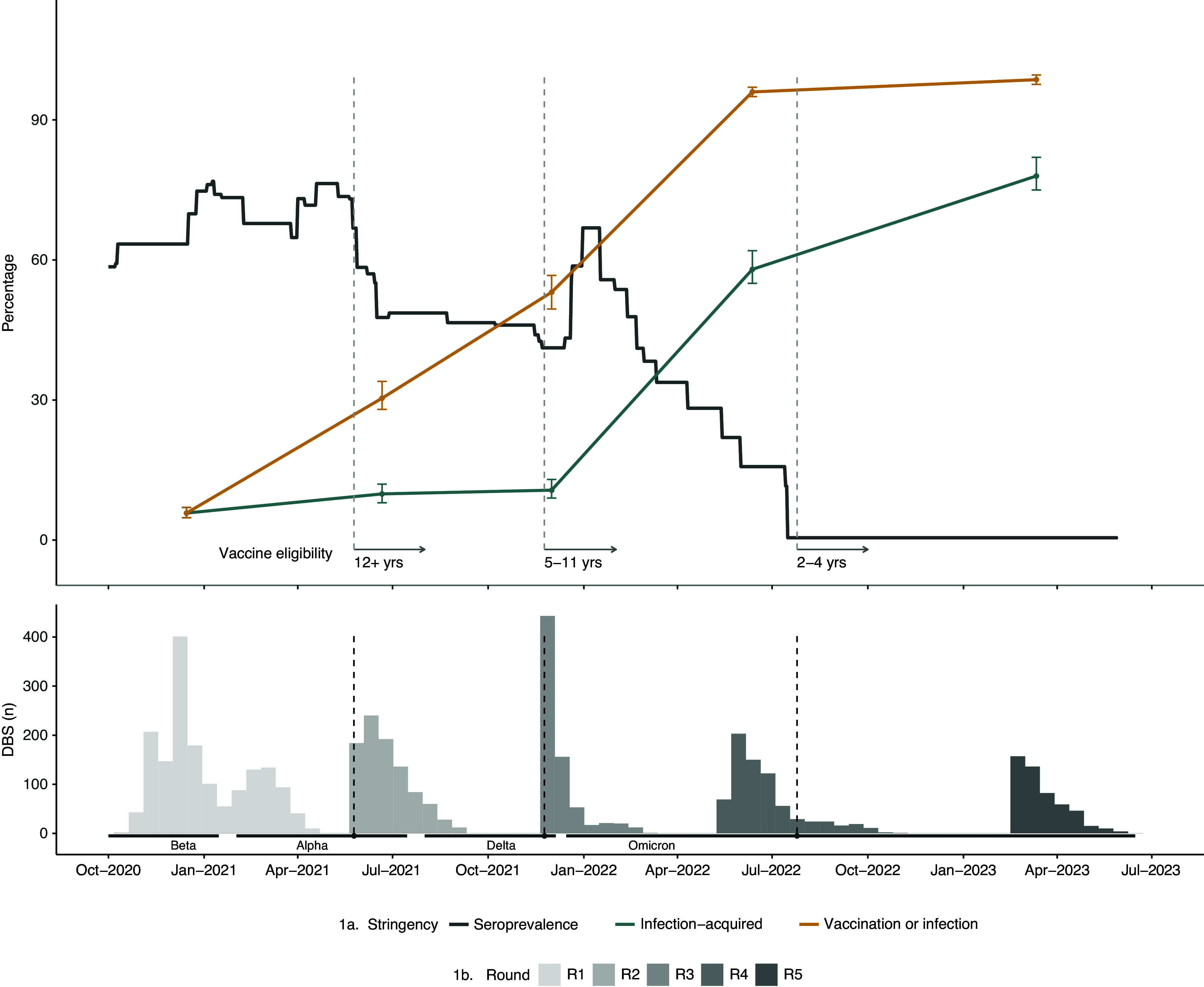
Seroprevalence among EnCORE participants across five rounds of data collection, highlighting the timing of DBS samples, age-specific vaccine eligibility dates, and periods dominated by variants of concern.

The vaccine and/or infection seroprevalence of 98% and the infection-acquired seroprevalence of 78% in our sample were comparable to the ~100% and 76%, respectively, reported in the general Canadian population at the beginning of 2023 [5], which is consistent with other studies that found slightly higher infection rates in younger children but lower vaccination rates in the general population [5]. In our study population, infection-acquired seroprevalence increased from 5.8% in late 2020 to 78% in early 2023 (Round 1 to 5 seroprevalence equal to 6%, 10.5%, 11%, 58%, and 78%, respectively; Figure 1) [1–2]. Though increased levels of antibodies are known to be correlated with a reduced risk of symptomatic infection [6], and the high seroprevalence in this study population is reassuring, it is unclear whether the levels of antibodies that correspond to a seropositive classification translate to a meaningful increase in protection from infection and whether this protection will persist in the face of the rapidly evolving Omicron variant.

We found that vaccinated participants had a lower risk of infection-acquired seropositivity though nearly a third had been vaccinated over a year before their DBS sample. Part of the higher seroprevalence in unvaccinated participants may be due to parents of children with a past infection and delaying or foregoing vaccination (reverse causality). Consistent with other studies [7], ethnic or racial minority status and lower income were associated with a higher risk of infection [1–2]. Though lower vaccine uptake in these demographic groups may be part of the explanation, these associations were also observed in the first round of data collection (October 2020 to April 2021) before vaccine eligibility. Furthermore, in the current round, the higher seroprevalence persisted when restricting the Round 5 analysis to vaccinated children. The reasons for this disparity are likely multifactorial and context-specific [8], involving factors such as household crowding, use of public transportation, and essential worker status, which are hard to address, from a public health perspective. However, any socio-demographic and socio-economic disparities in vaccination uptake in these groups may be mitigated by minimizing transportation and time barriers (e.g. wait times, inconvenient clinic hours) for residents of ethnically diverse and lower socio-economic communities, possibly through partnerships with communities and employers. In addition, culturally competent messaging may more effectively communicate the benefits of vaccination to diverse populations [8–9].

Surprisingly, after at least 7 months of follow-up (median: ~ 10.5 months), less than 14% of children newly infected in Round 4 were seronegative at their Round 5 test. In contrast, in rounds 1 to 3, the estimated time for approximately 50% of the sample to serorevert was 7.7 months [2]. However, it is likely that reinfection with Omicron, and possibly vaccination, reduced the percentage of seroreversions. Carazo et al. [10] found that healthcare workers with an Omicron BA.1 primary infection and at least two messenger ribonucleic acid (mRNA) vaccine doses were protected from new infection for at least 5 months [10]. In our study, though most participants remained seropositive for at least 10 months, we are uncertain of the duration of meaningful protection during that time. More research is needed regarding the duration of protection from infection in paediatric populations with high rates of infection [10].

In summary, we have seen a sharp increase in both overall seroprevalence and infection-acquired seroprevalence of SARS-CoV-2 in children, so that nearly all participants demonstrated some level of protection against symptomatic infection and severe outcomes from a future infection. However, children in lower-income households and those whose parents identify as an ethnic or racial minority continue to carry a greater risk that may be mitigated by greater vaccine uptake.

## Supporting information

Charland et al. supplementary material Charland et al. supplementary material

## Data Availability

Data are available on reasonable request from the corresponding author.

## Supplementary material

For supplementary material accompanying this paper visit http://doi.org/10.1017/S0950268824000797. click here to view supplementary material
