# Supplementary material for: Omicron incidence and seroprevalence among children in Montreal, Canada, in early 2023: final results from the longitudinal EnCORE serology study
Source: Epidemiol Infect. 2024 Sep 25;152:e103. doi: 10.1017/S0950268824000797 (PMC11427972; doi:10.1017/S0950268824000797)
Supplement: Charland et al. supplementary material [file S0950268824000797sup001.docx]

**Appendix: Supplementary Methods**

**Laboratory assays and validation protocols**

Participants’ serostatus was determined by enzyme-linked immunosorbent assays using the receptor-binding domain (RBD) from the spike protein, the full-length spike protein (S) and the nucleocapsid protein (N) as antigens. We validated each ELISA assay with positive control samples (participants with RT-PCR–confirmed SARS-CoV-2 infection and known to be seropositive for anti–SARS-CoV-2 antibodies) and negative control samples (SARS-CoV-2 seronegative). Based on the results, the RBD assay had a sensitivity of 95% and specificity of 100%, the S assay had a sensitivity of 100% and specificity of 100%, and the N assay had a sensitivity of 90% and a specificity of 95%. Colorimetric reactions determined by optic density (OD) allowed the detection of IgG in the samples and the evaluation of the signal generated by SARS-CoV-2–specific antibodies against each antigen confirmed whether subjects were seropositive. The OD cutoffs for positivity was determined based on the average of OD from negative sera plus 3 SDs.

**Serostatus determination**

If a participant was positive for any two of the three antigens, and was unvaccinated or received their first dose of a COVID-19 vaccine within 9 days of their dried blood spot (DBS) sample, they were classified as being infection-acquired seropositive ^1^ . If a participant received at least one dose of a COVID-19 vaccine at least 10 days prior to collecting their DBS sample and was positive for N and at least one other antigen, they were classified as being infection-acquired seropositive. Seropositivity by vaccination and/or infection was defined as being seropositive to at least two antigens.

**References**

1. Kanji JN, Bailey A, Fenton J, et al. Detection of SARS-CoV-2 antibodies formed in response to the BNT162b2 and mRNA-1237 mRNA vaccine by commercial antibody tests. Vaccine. 2021;39(39):5563-5570. doi:10.1016/j.vaccine.2021.08.022
